# Fibroblast growth factor-21 (FGF21) analogs as possible treatment options for diabetes mellitus in veterinary patients

**DOI:** 10.3389/fvets.2022.1086987

**Published:** 2023-01-09

**Authors:** Ronald J. Corbee, Dion L. van Everdingen, Hans S. Kooistra, Louis C. Penning

**Affiliations:** Department of Clinical Sciences, Faculty of Veterinary Medicine, Utrecht University, Utrecht, Netherlands

**Keywords:** glucose, metabolism, obesity, insulin, FGF, endocrine, diabetes

## Abstract

Fibroblast growth factors (FGFs) are involved in numerous metabolic processes. The endocrine subfamily of FGFs, consisting of FGF19, FGF21, and FGF23, might have beneficial effects in the treatment of diabetes mellitus (DM) and/or obesity. The analog with the greatest potential, FGF21, lowers blood glucose levels, improves insulin sensitivity, and induces weight loss in several animal models. In this review we summarize recent (pre)clinical findings with FGF21 analogs in animal models and men. Furthermore, possible applications of FGF21 analogs for pets with DM will be discussed. As currently, information about the use of FGF21 analogs in pet animals is scarce.

## Introduction

Diabetes Mellitus (DM), i.e., persistent hyperglycemia, is a very common endocrine disorder caused by various etiologies, with interesting differences between species. For example, in dogs DM is most frequently due to (immune-mediated) destruction of pancreatic beta-cells, but may also be due to excessive circulating concentrations of insulin antagonistic hormones, such as cortisol and (progesterone-induced) growth hormone (1). In contrast, the majority of diabetic cats has a type of DM that is very comparable with type 2 DM in humans, in which both insulin resistance (induced by lifestyle factors such as obesity and physical inactivity) and β-cell destruction play an important role in the pathogenesis (2–5), but DM in cats is also frequently caused by hypersomatotropism due to a pituitary tumor (6). Subcutaneous injections with insulin, usually twice daily, are almost always needed in the treatment of DM (7), which has an impact on the (quality of) life of both pets and owners (8, 9). A study from Niessen et al. with over a thousand veterinarians even showed that ~20% of cats diagnosed DM were euthanized within the first 2 years after diagnosis (9). Although remission of feline DM is possible, insulin administration in combination with dietary measurements and advices to increase physical activity quite often are insufficient to prevent hyperglycemic periods (7). Moreover, insulin treatment may also result in (life-threatening) hypoglycemic events (4). Consequently, drugs that may help to better regulate pets with DM or may result in diabetic remission are urgently needed. Fibroblast growth factor 21 (FGF21) analogs may be interesting in this respect.

**Figure 1:**
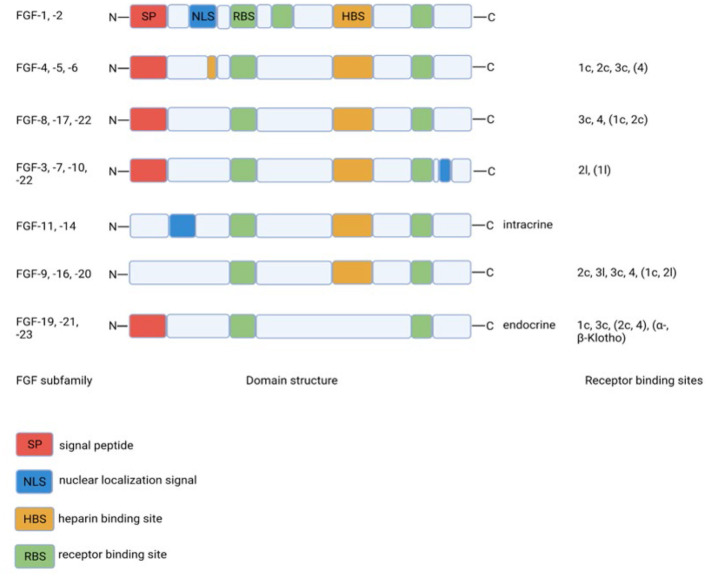
Structural homology between FGF-family members and binding to specific FGF-receptor subtypes. The lack of the heparin binding site in the endocrine FGF19 subfamily clearly discriminates this subfamily from the other FGF-family members. Created with BioRender.com.

Fibroblast growth factors are extracellular signaling molecules with a plethora of functions, ranging from inducing proliferation to effects on glucose and lipid metabolism (10, 11). Although the amino acid homology between the various FGF members is limited, the domain structure is much more conserved. The conserved core region consists of 12-stranded β-sheets in the center of the around 200 amino acid long protein (Figure 1). The lack of a heparin-binding site (HBS) clearly discriminates FGF19, FGF21, and FGF23 from the other FGFs. As a consequence, FGF19, FGF21, and FGF23 cannot bind to endothelial membrane-bound receptors directly and this allows them to diffuse easily through the bloodstream. Therefore, FGF19, FGF21, and FGF23 do not act in an autocrine/paracrine fashion but function more as hormones in an endocrine mode of action. Binding to target cells requires the co-expression of α-Klotho or β-Klotho in addition to the classical FGF-receptors. The specific tissue expression of α-Klotho (for FGF23) and β-Klotho (for FGF19 and FGF21) explains why FGF23 acts upon the kidney/bone and FGF19 and FGF21 act on the liver/gallbladder and liver/adipose tissue, respectively (Table 1). Because of their effects on liver and adipose tissue, especially FGF19 and FGF21 may be interesting with regard to the treatment of diabetes mellitus.

**Table 1:** Overview of the FGF19 family members.

	**FGF19**	**FGF21**	**FGF23**
Subtypes of FGF-receptor binding	β-klotho, 1c, 4	β-klotho, 1c, 3c	α-klotho, 1c, 3c, 4
Produced in	The intestines	The liver	Bone
Affects	Liver, muscle, adipose tissue, CNS	Liver, muscle, adipose tissue, pancreas, CNS	Heart, intestines, kidney, bone
Effects	+ Glycogen synthesis	+ β-oxidation	+ Renal sodium excretion
	– Gluconeogenesis	+ Insulin sensitivity	– Vitamin D3 synthesis
	– Bile acid synthesis	+ TCA levels (12)	
	– Lipogenesis		

FGF19 was discovered in 1999 from a human fetal cDNA library, based on the homology with probes for mouse FGF15 (13). The deduced amino acid sequence had 51% homology with the mouse FGF15. Upon binding to β-Klotho in conjunction with FGF-R1c/4, FGF19 stimulates hepatic glycogen synthesis and inhibits gluconeogenesis via its target genes glucose-6-phosphatase (G6Pase) and phosphoenolpyruvate carboxykinase (PEPCK). Furthermore, it inhibits bile acid production via the inhibition of CYP7A1 expression, a crucial enzyme in bile acid synthesis (14–16). Lastly, FGF19 inhibits hepatic lipogenesis.

One year after the discovery of FGF19, the same group identified FGF21, which had only 35% sequence identity with FGF19 (17). In adipose tissue, FGF21 increases the expression of GLUT1 (glucose transporter), thus facilitating glucose uptake (18). Serum and hepatic triglyceride levels were lower in FGF21-transgenic mice (19). This process is dependent on the expression of CD36, which is responsible for free fatty acid uptake (20). Finally, the metabolic effects of FGF21 include an increase in β-oxidation via increased expression of 3-hydroxyacyl-CoA dehydrogenase, carnitine palmitoyltransferase (CPT), peroxisome proliferator-activated receptor-gamma coactivator 1-α (PGC-1-α), and acyl-CoA oxidase (21).

The *in vitro* effects of FGF19 and FGF21 on metabolism suggest beneficial effects of FGF19 and FGF21 in the treatment of DM *in vivo*. The biological half-life of FGF19 is around 30 min, whereas FGF21 has a half-life of 2 h at maximum (22), which poses challenges in case these compounds are to be implemented in clinical settings. Therefore, analogs with enhanced stability have been developed. The longer half-life of FGF21 compared to FGF19 is most likely one of the reasons why most pharmaceutical companies have focused on the development of FGF21 analogs (23–26).

Most studies with the FGF21 analogs have been conducted in mice, monkeys or humans; studies in pets are scarce. Because the FGF21 analogs may have potential in the treatment of pets with DM, the aim of this study was to review the recent literature regarding the use of FGF21 (analogs).

## Effects of FGF21 on metabolism

FGF21 has emerged as the preferred therapeutic target FGF for metabolic diseases such as non-alcoholic steatohepatitis (NASH), metabolic syndrome, and diabetes mellitus (DM). The preference for FGF21 over FGF19 and FGF23 is based on several observations. First, its preferential liver expression. Second, recombinant FGF21 stimulates glucose uptake in mouse 3Te-L1 adipocytes and primary human adipocytes and not in non-adipocytic cells (27). This observation prompted this group to investigate FGF21-effects in *ob/ob* and *db/db* mice, two classical models for obese and diabetic mice, respectively (27). FGF21 reduced plasma glucose and triglyceride concentrations to normal values up to 24 h after supplementation. GLUT1 mRNA and protein levels were increased by FGF21. Importantly, FGF21 did not induce hypoglycemia, cell proliferation, or increase body weight. These observations clearly established the potential of FGF21 as a regulator of aspects of the metabolic disturbances in obesity, NASH, and DM.

Subsequently, other metabolic improvements induced by FGF21 were reported such as a reduction of body weight, protection against high-fat diet-induced steatosis (lipid accumulation in hepatocytes), and improved serum cholesterol and insulin concentrations (19, 28–32). FGF21 stimulates the transformation of stem cells to adipocytes (18). FGF21 requires mature adipocytes to exert its metabolic effects. Fasting and a ketogenic diet induce FGF21 expression (19, 33). This enhanced expression is mediated via peroxisome proliferator-activated receptor-alpha (PPAR-α) (15, 27, 34, 35). FGF21 levels are also increased in obese individuals and in patients with NASH (36). Apparently, this increase is not sufficient to revert the steatotic liver phenotype, despite the fact that FGF21 stimulates β-oxidation (37, 38). This enhanced β-oxidation is mediated via increased expression of hepatic protein (PGC-1α), resulting in lower plasma and hepatic lipid levels (12, 28, 29).

Part of the effects of FGF21 are mediated by PPAR-γ (30). Rosiglitazone is a stimulator of the PPAR-γ pathway which has been studied in conjunction with FGF21; the effects of FGF21 and rosiglitazone were studied both individually and combined. Apart from their own effects, a synergistic effect could be demonstrated, which was a 10 fold higher expression of the insulin-independent GLUT1 in adipose tissue, as well as increased glucose uptake (18).

Studies showed a significant decrease in obesity in monkeys and mice upon FGF21 treatment. Furthermore, FGF21 decreased the number of VLDL-receptors (very low density lipoproteins) in the liver. These receptors transport fatty acids into the liver (39). Inhibition of this uptake leads to less fat in the liver and therefore less steatosis. FGF21 is known to increase insulin sensitivity in the liver. This increase could lead to more glycogen storage in the liver and more glucose uptake.

FGF21 represses the mammalian target of rapamycin complex 1 (mTORC1) in mice. This is a critical regulator in the pathogenesis of DM. FGF21 could therefore improve insulin sensitivity (40). This higher insulin sensitivity could also be the consequence of improved pancreatic β-cell function. Indeed, diabetic rodents had more glucose-induced insulin secretion upon FGF21 treatment (41).

One of the major effects of FGF21 is lowering hyperglycemia. This effect is mainly caused by increased glucose uptake, mostly by adipose tissue, as well as by reduced gluconeogenesis in the liver (27). The increased glucose uptake is thought to be the consequence of an increased expression of GLUT1, specifically in adipose tissue, as demonstrated in mice (18, 27, 42). Moreover, FGF21 combined with insulin sensitizes brown adipose tissue (BAT) for more glucose uptake, which is used for heat production, thus lowering blood glucose levels (43). FGF21 knockdown in mice showed an increase in both glycogenolysis and gluconeogenesis, indicated by increased G6Pase and PEPCK levels, suggesting that FGF21 has inhibitory effects on gluconeogenesis and glycogenolysis (44).

FGF21 is known to have weight losing potential by increasing energy expenditure in BAT. Mice receiving FGF21 supplements demonstrated higher oxygen intakes, lower respiratory quotients [*viz*. enhanced fat oxidation (45)], and small elevations of the core body temperature (28, 29, 46). Increased β-oxidation can contribute to weight loss and consequently reduce obesity development. The type of β-oxidation is BAT-mediated by uncoupling protein 1 (UCP1), which uncouples respiration from heat production. This means that no ATP is produced by the oxidation of fat, but the energetic fatty acids are merely used to produce heat (47). Moreover, FGF21 induces the expression of UCP1 in white adipose tissue (WAT), the so-called “browning process” which leads to enhanced thermogenesis, without ATP production, upon fat oxidation (48).

Altogether, FGF21 is clearly a key metabolic regulator in animals. It supports the stabilization of normoglycemic states, prevents the liver from steatosis, and could reduce the risk for obesity. Pets with DM present a challenge to regulate glycemic and lipid levels, and obesity. Therefore, it is conceivable to observe positive effects of FGF21 in diabetic animals.

### FGF21 analogs in humans and animal models with DM

Based on the longer half-life of FGF21 compared to FGF19, and the metabolic effects of FGF21, FGF21 and more stable analogs have gained attention of several pharmaceutical and biotechnological companies like Lilly Research Laboratories (LY2405319), Pfizer (PF05231023), and Bristol-Myers Squibb [(PEG-)cFGF-21] to interfere in metabolic diseases. Each of these FGF21 analogs or stabilized constructs will be described with the emphasis on glucose and insulin lowering capacity, body weight reduction potential, and improvement of lipid profiles. This data is summarized in Table 2.

**Table 2:** Summary of species-specific findings of FGF21 analogs.

**FGF21 analog**	**Weight loss**	**Insulin levels ↓**	**Glucose levels ↓**	**[Lipid] improvement**
LY (40, 49, 50)	• MI & MO: yes • HU: no	In all: yes	• MI & MO: yes • HU: no	HU & MO: yes
PF (22, 51, 52)	• MO: yes • HU: no	• MI: yes • Rest: no	• MI: yes • Rest: no	In all: yes
RGE (53)	MI and MO: yes	In all: yes	In all: yes	In all: yes
(PEG-)cFGF21 *Dog only* (40)	Yes	*	Yes	Yes

[Lipid] improvement: improved lipid levels in the blood.

^*^ = insulin levels in dogs with primary DM are already very low.

MI, mice; MO, monkeys; HU, humans.

Naive FGF21 is not suitable to use within pharmaceutical preparations as it is a biophysically and thermally unstable protein. For that reason, a more potent variant of FGF21 was produced by DNA modification (LY2405319), which was proven to be much more stable and therefore more potent for pharmacological use in animals (54).

LY2405319 has been tested in mice (54), monkeys (49), and humans (50). The human patients were experiencing diabetes for an average of 7 years, whereas in the other species the obese and diabetic conditions were experimentally induced. In monkeys and humans, there was a significant improvement in fat composition in the blood on LY2405319 treatment, as triglycerides and total cholesterol/LDL-cholesterol decreased. This has not yet been studied in mice.

In mice and monkeys, there was significant weight loss, which was reversed within the wash-out periods in monkeys. The mice showed no change in total food intake, suggesting an intrinsic weight losing effect of LY2405319. In humans, there was a trend for weight loss, however food intake was not recorded. Therefore, further research is necessary to unravel the underlying mechanism of the observed weight loss effect. This could be psychologically driven by the central nervous system (CNS), due to a change in fat composition, or both.

Mice and monkeys showed significant decreases in blood glucose levels. High doses of LY2405319 did not induce hypoglycemia, which is an advantage compared to the current treatment with insulin, as overdosing insulin can result in hypoglycemic coma and death. In humans, only a trend toward normoglycemia was noted on LY2405319 treatment. Insulin levels did drop in all studies, suggesting that insulin resistance decreased as a result of LY2405319 treatment. This drop of insulin may also improve β-cell repair, however this has not yet been studied.

Antibodies against LY2405319 were demonstrated and analyzed in the monkeys, however the effects of LY2405319 were still seen throughout the study period, suggesting that the antibodies were not neutralizing. One monkey with an advanced stage of diabetes was included. It had extremely low levels of insulin, severe hyperglycemia, and low body weight. Treatment with LY2405319 did not result in normoglycemia, only a small, but not sufficient trend in glucose reduction was observed. Insulin levels had a small increasing trend. A possibility could be that insulin and LY2405319 work synergistically; one needs the other, and therefore there was only a small trend toward normoglycemia. However, this monkey did show positive changes in lipid composition, which is encouraging in the treatment of advanced diabetic patients.

In humans, treatment with LY2405319 did not show any side effects, however the need for daily injections is a limitation for its use, although it is only once daily compared to twice daily insulin injections. In conclusion, LY2405319 has promising possibilities for DM treatment, but further research is warranted.

#### PF05231023

A more recent FGF21 analog, PF05231023, consists of two modified FGF21 molecules, conjugated to an antibody. PF05231023 has been tested in mice (50), humans (55), monkeys (51, 52, 55), and rats (51). All these studies demonstrated lipid lowering effect. The monkeys and humans showed a decrease in triglycerides and an increase in HDL. The humans in the two different studies had some differences in outcome. One study showed a significant decrease in total cholesterol and LDL (51), whereas in the other study no significant changes were seen (56). This could be the result of a different injection rate, which was either once or twice weekly.

Monkeys had a significant lower caloric intake, resulting in a lower body mass (52). In addition, fewer pro-inflammatory markers were measured, indicative of reduced inflammation. In contrast, a study in rats demonstrated increased food intake on PF05231023, but no changes in body mass. The authors attributed this due to higher energy expenditure on FGF21. These rats got their injection intracerebroventricular, which suggests that FGF21 acts predominantly within the brain (56). However, the action of FGF21 on the CNS is still unclear, and subject for further research.

A human study with once weekly injections gave conflicting results. Triglyceride levels were significantly lowered, but inconsequently no changes in body mass were observed. Only the study in mice showed significant changes in glucose and insulin levels.

Some adverse effects were reported in humans and rats. Diarrhea was seen in about 30% of humans, which was accompanied by increased bowel movements. Some individuals showed an increase in bone markers that were indicators of bone resorption. This side effect was unexpected as to date, FGF23 and not FGF21 has been regarded as a bone-remodeling factor. Furthermore, humans and rats showed an increase in heart rate and blood pressure. Blood pressure went up with a maximum increase of 15 Hg, which is potentially harmful in DM patients with already high blood pressure. Mice showed sympathetic nerve activity in response to FGF21 injections. This could be the explanation for the increase in blood pressure and heart rate because of the stimulation of the sympathetic nervous system (57).

PF05231023 was injected once or twice a week, which is an advantage of PF05231023 when compared to LY2405319 which was used comparable to insulin in a twice daily regimen. However, PF05231023 was injected intravenously in these studies, not subcutaneously as LY2405319.

To conclude, PF05231023 has promising effects on the lipid metabolism profile and body mass. Unfortunately, no effect on blood glucose levels was seen. Side effects such as diarrhea, bone resorption, and increased heart rate and blood pressure have been reported in the studies, which is one of the reasons why PF05231023 treatment needs to be further optimized.

#### Fc-FGF21(RGE)

Fc-FGF21(RGE) is a novel FGF analog, with improvements made compared to their earlier analog [Fc-FGF21(RG)]. By this modification, there was less aggregation, proteolytic degradation, and a better affinity for the β-klotho receptor, allowing for once or twice weekly subcutaneous injections. RGE has been tested in mice and monkeys (53). Both species showed a decrease in glucose levels, insulin levels, triglycerides, and body weight, all of which were significantly better compared to the previous analog. Mice and monkeys had also a reduced food intake, which could be the reason for the observed weight loss.

Because of the better pharmacokinetics and -dynamics, RGE could be one of the most promising analogs. Moreover, no hypoglycemic episodes were reported in this study. The half-life of RGE was also increased compared to the previous analog. Future studies on the use of RGE should be performed in humans or pets to see if similar results can be obtained.

#### (PEG)-cFGF21

To our knowledge, the first long-during study in diabetic dogs with an FGF21 analog was performed with PEGylated canine FGF21 (PEG-cFGF21). This was done in dogs who present primary DM, characterized by immune-mediated destruction of the pancreatic β-cells (58). PEG and cFGF21 were both able to lower glucose levels. Compared to treatment with insulin, which was able to maintain glucose levels normal for 2 h, cFGF21 and PEG maintained it for, respectively, 2 and 3 days. PEG had stronger effects compared to cFGF21 over time, suggesting PEG to be the better long-acting analog.

Triglyceride, total cholesterol, and LDL-levels were decreased in both analog groups, when compared to insulin. Furthermore, GLUT1 expression was increased in the liver, which could be indicative of concurrent increased expression of GLUT1 in adipose tissue which could be beneficial for DM patients. Moreover, expression of sodium-glucose cotransporter 1 (SGLT1) and SGLT2 decreased significantly. These transporters are used for (re)absorption of glucose from out of the kidneys (SGLT1) and intestines (SGLT2), back into the bloodstream. Fewer of these receptors results in lower blood glucose levels, which is beneficial in hyperglycemic patients.

The pancreas had decreased numbers of reactive oxygen species (ROS) and inflammatory markers, which points to less severe damage of the pancreas in diabetic patients. PPAR-γ was upregulated in adipose tissue, which could also lead to improved insulin sensitivity. The question remains whether these results in canine primary DM can be translated to cats with secondary DM, but the prospects on insulin levels are good and no adverse effects were reported so far.

## Future perspectives

Before application of FGF21 in clinical practice, more research should be done on the underlying mechanism in diabetic patients such as regulation of SGLT in the kidneys, and the effect of FGF21 on the central nervous system, possible synergistic effects with insulin, long term effects, different application forms (oral, parenteral), and possible side effects. Ideally, these mechanisms and effects should be studied in clinical trials with the intended target species.

## Final remarks

Extrapolation of results based on human and rodent, species that are omnivores, to the adaptive carnivores (i.e., dogs and cats) has some limitation especially regarding glucose and lipid homeostasis. Where men/mice have *de novo lipogenesis* (DNL) in the liver, in cats this occurs predominantly in fat tissue (59). This is probably due to the fact that cats are more strict carnivores whereas dogs are considered adaptive carnivores that are more used to thrive on an omnivorous diet, just like men and mice. Moreover in cats reduced food uptake will lead to recruitment of lipids from the fatty tissue to the liver. As the feline liver sometimes cannot cope with this sudden increase of lipid mobilization, even in a situation of starvation, cats may develop hepatic lipidosis. Dogs are also better able to cope with differences between nutrient abundance and starvation due to the feast and famine eating pattern of their ancestors (i.e., wolves). This sharply contrasts with mice and men. The details of the mechanistical species differences include the already mentioned differences in DNL and PPAR-activities.

In conclusion, FGF21 (and its analogs) has multiple favorable effects on metabolism, especially in humans with secondary DM. Paradoxically, FGF21 levels are elevated in obese or diabetic animals (60–62). When these obese mice were given more FGF21, they did not fully respond anymore. Two explanations can be made in this paradox. FGF21 could be harmful to the body and its metabolism, or obese patients could have a resistant state for FGF21. If FGF21 should have destructive effects, why did FGF21 demonstrate positive in almost all the animals in previous studies? Furthermore, FGF21 is physiologically present in the body, and therefore not likely to be harmful. The resistance hypothesis is thus more plausible. FGF21 was highly increased in mice that were fed a ketogenic diet. β-klotho- and FGFR1-receptors were reduced expressed in adipose tissue, whereas in the liver FGFR4-receptors were reduced expressed, with no change in β-klotho-receptor expression. Both observations led to an impaired signaling of FGF21 in ketogenic fed mice (33). This was also observed in obese mice, where the expression of β-klotho- and FGFR1-receptors reduced in adipose tissue, after a few weeks with the increasing level of obesity (63). On the contrary, overexpression of β-klotho receptors leads to more weight loss in obese animals and increased sensitivity to FGF21 (38).

Altogether, FGF21 might play a role in the treatment of pets with DM in the future, eventually combined with exercise instructions and dietary adaptations. FGF21 can tackle several important aspects of DM, by increasing insulin sensitivity, reducing hyperglycemia, improving the lipid metabolism profile of the blood, and inducing weight loss. To be a good alternative for, or an addition to insulin therapy, limitations and side effects should be reduced.

In 1922, insulin was firstly described by Banting and Best based on canine studies (64). It looks like one century later, a novel step to tackle DM is on the horizon.

## Author contributions

DLvE drafted the manuscript as part of a BSc-thesis. RJC adapted this manuscript to the journals' requirements. RJC, HSK, DLvE, and LCP edited and supervised this manuscript. All authors contributed to the article and approved the submitted version.
